# Responses of key root traits in the genus *Oryza* to soil flooding mimicked by stagnant, deoxygenated nutrient solution

**DOI:** 10.1093/jxb/erad014

**Published:** 2023-01-11

**Authors:** Shuai Tong, Johan Emil Kjær, Lucas León Peralta Ogorek, Elisa Pellegrini, Zhiwei Song, Ole Pedersen, Max Herzog

**Affiliations:** Department of Biology, University of Copenhagen, Universitetsparken 4, 3rd floor, 2100 Copenhagen, Denmark; Department of Biology, University of Copenhagen, Universitetsparken 4, 3rd floor, 2100 Copenhagen, Denmark; Department of Biology, University of Copenhagen, Universitetsparken 4, 3rd floor, 2100 Copenhagen, Denmark; Department of Biology, University of Copenhagen, Universitetsparken 4, 3rd floor, 2100 Copenhagen, Denmark; Department of Agricultural, Food, Environmental and Animal Sciences, University of Udine, Via delle Scienze 206, 33100 Udine, Italy; Department of Biology, University of Copenhagen, Universitetsparken 4, 3rd floor, 2100 Copenhagen, Denmark; Department of Biology, University of Copenhagen, Universitetsparken 4, 3rd floor, 2100 Copenhagen, Denmark; School of Agriculture and Environment, The University of Western Australia, 35 Stirling Highway, WA 6009, Australia; Department of Biology, University of Copenhagen, Universitetsparken 4, 3rd floor, 2100 Copenhagen, Denmark; Lancaster University, UK

**Keywords:** Aerenchyma, barrier to radial oxygen loss, phenotypic plasticity, radial oxygen loss, radial water loss, rice, root porosity, root respiration, waterlogging

## Abstract

Excess water can induce flooding stress resulting in yield loss, even in wetland crops such as rice (*Oryza*). However, traits from species of wild *Oryza* have already been used to improve tolerance to abiotic stress in cultivated rice. This study aimed to establish root responses to sudden soil flooding among eight wild relatives of rice with different habitat preferences benchmarked against three genotypes of *O. sativa*. Plants were raised hydroponically, mimicking drained or flooded soils, to assess the plasticity of adventitious roots. Traits included were apparent permeance (*P*_A_) to O_2_ of the outer part of the roots, radial water loss, tissue porosity, apoplastic barriers in the exodermis, and root anatomical traits. These were analysed using a plasticity index and hierarchical clustering based on principal component analysis. For example, *O. brachyantha*, a wetland species, possessed very low tissue porosity compared with other wetland species, whereas dryland species *O. latifolia* and *O. granulata* exhibited significantly lower plasticity compared with wetland species and clustered in their own group. Most species clustered according to growing conditions based on *P*_A_, radial water loss, root porosity, and key anatomical traits, indicating strong anatomical and physiological responses to sudden soil flooding.

## Teaser

Soil flooding resulted in strong acclimation in key root traits for three cultivars and six wild species of rice, while two dryland species showed little plasticity in root traits.

## Introduction

Ongoing global climate change has already resulted in more frequent and severe flood and drought events. Agricultural crops, including rice, are affected by a suite of climate-related variables known to act as abiotic stressors, e.g. too much or too little precipitation as well as temperature extremes (Weigel, 2005). Floods not only cause tragic losses to humans, but they also damage crops (Pedersen *et al.*, 2017) with disastrous examples from many parts of the world (Bailey-Serres *et al.*, 2012). Therefore, novel climate-smart cultivars with resilience to abiotic stress are urgently needed to improve the world’s food security (Lipper *et al.*, 2014). Compared with its wild relatives, cultivated rice has a low genetic diversity, and using germplasm from wild relatives to improve rice is therefore a promising strategy to improve genetic diversity and resilience of cultivated rice (Brar and Khush, 2018). To promote this process, we phenotyped eight wild relatives of rice from the *Oryza* genus and three genotypes of *Oryza sativa* (cultivated Asian rice) for root traits conferring soil flooding tolerance. We also established a plasticity index for key root traits showing the ability of rice species to acclimate to soil flooding.

Lowland rice, unlike other cereals, thrives in paddy fields and is therefore generally tolerant to soil flooding (Nishiuchi *et al.*, 2012). Soils of flooded paddy fields are anoxic since the slow diffusion of O_2_ from the atmosphere is insufficient to meet the O_2_ consumption by plant roots and soil organisms (Ponnamperuma, 1984). The anoxic soil environment poses a range of challenges to rice roots, and a suite of root traits have thus evolved in the ancestors of cultivated rice to help cope with the hostile soil environment. The challenges caused by anoxic soils can be divided into two main components: (i) lack of O_2_ can result in severe root tissue hypoxia (or even anoxia) (Armstrong, 1979) since O_2_ for respiration cannot be obtained by radial inward diffusion from the external soil, and (ii) lack of molecular O_2_ in the soil results in a switch to alternative electron acceptors by the microbial community, and the resulting waste products from microbial fermentation can be extremely toxic to plant roots (Ponnamperuma, 1972).

Supply of molecular O_2_ to fuel respiration of roots in anoxic soils can be achieved via a number of key root traits. One such key trait is high tissue porosity owing to intercellular gas-filled spaces and aerenchyma, the gas spaces representing a low resistance pathway for longitudinal molecular diffusion of O_2_ from shoot tissues to root apices (Armstrong, 1979). In flooded soils, high root porosity results in deeper rooting, and the root porosity of some wetland plants can reach 55% due to extensive formation of aerenchyma (Colmer, 2003b). Aerenchyma is constitutively formed in roots of rice but is further enhanced by inducible aerenchyma as a response to soil flooding. Constitutive aerenchyma enables survival of roots at the onset of soil flooding, whereas inducible aerenchyma is needed to further enable root penetration into the anoxic soils (Pedersen *et al.*, 2021b). Another key root trait conferring tolerance to anoxic soils is the root barrier to radial O_2_ loss (ROL). Some wetland plants can form a barrier to ROL in the basal zones of the roots, and the barrier significantly reduces O_2_ loss via radial diffusion to the surrounding anoxic soil (Colmer, 2003b). Consequently, longitudinal O_2_ diffusion in the aerenchyma towards the root apex is greatly enhanced (Colmer, 2003b). The root barrier to ROL of cultivated rice is induced as a response to soil flooding, whereas it is constitutive in some species of wild rice (Ejiri *et al.*, 2020). The barrier is formed in the outer cell layers and is thought to mainly consist of suberin deposits in the exodermis (Kulichikhin *et al.*, 2014). The strength of the barrier varies among species and rice cultivars and has been classified into three categories: ‘tight’, ‘partial’, and ‘weak’ (Colmer, 2003b). Finally, thick roots and a high cortex-to-stele ratio (CSR) also enhance tissue internal aeration in flooded soils. Thick roots act by reducing the surface area to volume ratio so that the contact area to the anoxic soil per volume of tissue is smaller; this reduces the tendency for O_2_ to diffuse into the anoxic environment (Pedersen *et al.*, 2021b). A high CSR reduces root respiration since stelar tissues are dense and metabolically active, whereas the cortex is of high gas-filled porosity (Yamauchi *et al.*, 2021); a reduced O_2_ consumption per unit of root length saves O_2_ so that root penetration is enhanced. The above key root traits are all essential for growth in flooded soils, but plasticity of these traits in species of wild rice should be evaluated to determine if these species can be used to further improve tolerance to flooded soils in cultivated rice.

The root ROL barrier is possibly a more essential trait than previously thought. In addition to its well described function in enhancing tissue O_2_ status, it may also protect against soil phytotoxins (Jiménez *et al.*, 2021b). When molecular O_2_ is absent, soil microbes utilize alternative electron acceptors elevating the concentration of certain harmful substances in the soil. Reduced forms of manganese (Mn^2+^) and iron (Fe^2+^) in addition to H_2_S and low molecular mass carboxylic acids frequently increase in flooded soils (Ponnamperuma, 1972). These toxic substances can potentially enter the roots with adverse effects on root respiration and growth (Mongon *et al.*, 2014; Colmer *et al.*, 2019). However, the root barrier to ROL restricts apoplastic diffusion of reduced iron, preventing accumulation to toxic levels (Jiménez *et al.*, 2021b). A beneficial role in restricting intrusion of H_2_S or carboxylic acids has not yet been experimentally demonstrated, but the barrier restricts radial diffusion of molecular hydrogen as well as of water vapour (Peralta Ogorek *et al.*, 2021), suggesting a potential role in also restricting intrusion of toxic gases from flooded soils.

Plants are sessile and therefore they must be able to respond when soil flooding occurs or disappears, and such responses are referred to as ‘phenotypic plasticity’ (Brooker *et al.*, 2022). Most responses to soil flooding are plastic, involving formation of additional aerenchyma and adventitious roots near the soil surface (Justin and Armstrong, 1987; Visser *et al.*, 1996; Cox *et al.*, 2003). However, traits involved in soil flooding tolerance vary greatly among species, with significant changes in expression, amplitude, and timing of responses (Grimoldi *et al.*, 2005). Similarly, responses to soil flooding may vary in the *Oryza* genus, where dryland species such as *O. granulata* are expected to show little plasticity in root traits related to soil flooding whereas a wetland species such as *O. longistaminata* is hypothesized to show higher plasticity. The relative distance plasticity index (RDPI) is a numerical index that can be used to quantify phenotypic plasticity of multiple quantitative root traits in ­response to soil flooding, allowing statistical comparisons among species (Valladares *et al.*, 2006). We therefore aimed to combine key root traits involved in soil flooding tolerance into a single number, to characterize plasticity of each species and genotype.

Overall, we aimed to identify candidate species for soil flooding tolerance in a range of laboratory-grown wild relatives of rice and landraces and to reveal diversity in soil flooding-related traits by combining diagnostic anatomical and physiological tools using state-of-the-art microsensors. Eight wild relatives of rice and three *O. sativa* genotypes were grown hydroponically under aerated or stagnant, deoxygenated conditions after which root thickness, CSR, root porosity, ROL barrier strength and apoplastic barriers in the exodermis were measured in newly formed adventitious roots. Rates of radial water loss from roots were measured to test the extent to which the ROL barrier also restricts water loss. We tested the hypothesis that wild relatives of rice from dry habitats would have lower root porosity, lower CSR and low ROL barrier strength compared with species from wetlands, including domesticated rice. In other words, wild relatives of rice from wetland areas are hypothesized to exhibit higher phenotypic plasticity for soil flooding traits than dryland species.

## Materials and methods

### Plant material

Eight wild relatives of rice and three genotypes of *O. sativa* were used in this study (Table 1). Seeds were provided by the International Rice Research Institute (IRRI, Philippines), with the exception of NERICA-1, which was obtained from Sokoine University of Agriculture, Tanzania.

**Table 1.** List of the *Oryza* species included in the study

Species	Accession ID	Genome	Natural habitat^*a*^
*O. sativa *‘FR13A’	N/A	AA	Indian landrace and donor of *SUB1*^*b*^
*O. sativa *‘IR42’	N/A	AA	Moderately drought and submergence tolerant^*c*^
*O. glaberrima *× *O. sativa *‘NERICA-1’	N/A	AA	No phenotypic information
*O. barthii*	IRGC 86481	AA	In deep water, slowly flowing water, stagnant water and pools, seasonally flooded land
*O. glumaepatula*	IRGC 101960	AA	Swamps and marshes; usually deep water
*O. longistaminata*	IRGC 81960	AA	Swampy areas, edges of lakes and ponds, in and edge of rice fields, permanently wet or seasonally dry. Also deep water, but usually <1 m water depth
*O. nivara*	IRGC 86459	AA	Swampy areas, seasonally wet, shallow water
*O. latifolia*	IRGC 102481	CCDD	Wet or damp sites, in or near water, riverbanks, streams, or pool edges. In forests, fields, grasslands, swamps
*O. australiensis*	IRGC 86534	EE	Wet sites, extending further from sites of permanent water than other perennial *Oryza* thanks to rhizome helping to survive the dry season
*O. brachyantha*	IRGC 104155	FF	In rock pools, near streams, in water up to 0.5 m deep, but more often in shallow water
*O. granulata*	IRGC 102117	GG	Forest floor, damp and seasonally dry sites. Not found in standing water

The table shows species name or cultivar along with accession ID, type of genome, and habitat preferences. Species and genotypes are ordered according to genomes of AA, CCDD, EE, FF, and GG. N/A, not available.

^*a*^ Vaughan (1994), Menguer *et al.* (2017).

^*b*^ Ismail *et al.* (2013).

^*c*^ Ponnamperuma (1979).

For germination, seeds were imbibed for 3 h in aerated 0.5 mM CaSO_4_ before being transferred to a Petri dish with tissue paper moistened with 0.5 mM CaSO_4_; these were stored at 28 °C in the dark for 3–14 d depending on species. The seedlings were subsequently moved to a mesh floating on an aerated 25% strength nutrient solution (100% strength nutrient solution composition below) in a 14/10 h light–dark cycle for 7 d. The seedlings were then transferred to 3.6-litre pots (6-litre pots for the *O. longistaminata* and *O. latifolia* as these grew very tall) filled with full-strength nutrient solution and fixed to the pot lids using sliced 20 mm foam plugs. The full-strength nutrient solution contained (in mM): K^+^, 5.95; Ca^2+^, 1.5; NH_4_^+^, 0.625; Mg^2+^, 0.4; Na^+^, 0.2; NO_3_^−^, 4.375; SO_4_^2−^, 1.905; H_2_PO_4_^−^, 0.2; SiO_2_^2−^, 0.1; Mn^2+^, 0.002; Zn^2+^, 0.002; Ni^2+^, 0.001; Cu^2+^, 0.0005; Cl^−^, 0.05; BO_3_^3−^, 0.025; MoO_4_^2−^, 0.0005; FeEDTA, 0.05; MES buffer, 2.5 (pH adjusted to 6.0 using KOH, with this K^+^ included in the value stated above). The nutrient solution was constantly purged with air at the rate of 100 ml min^−1^, and the nutrient solution was renewed weekly. Plants were grown in a growth chamber at 30/25 °C; 14/10 h, light (250–450 µmol m^−2^ s^−1^)–dark cycle with relative humidity >90%, and watered with deionized (DI) water as needed. The plants were cultivated in aerated solutions for 3–4 weeks after which measurements of apparent permeance to O_2_, root respiration, radial water loss, root dimension, root porosity, and permeability of the root apoplast were conducted within 1–2 weeks using adventitious roots formed in these growing conditions.

After all of the measurements were conducted under aerated conditions, the plants were transferred to a stagnant, deoxygenated nutrient solution to mimic a sudden event of soil flooding. Prior to transfer to the stagnant, deoxygenated nutrient solution, plants received a hypoxic pre-treatment by purging the nutrient solution with gaseous N_2_ for 5 min and were then left overnight before being transferred to the stagnant, deoxygenated nutrient solution. The purpose of the pre-treatment is to prevent an anoxic shock (Gibbs and Greenway, 2003). The stagnant, deoxygenated nutrient solution had an identical composition to the aerated nutrient solution, but with 0.1% (w/v) agar added and was purged with gaseous N_2_ for 3 h. The inclusion of agar in the nutrient solution prevents convection and the consequent reintroduction of atmospheric gases, and it mimics the gas composition (very low O_2_ and elevated ethylene and CO_2_) characteristic of flooded soils (Wiengweera *et al.*, 1997). After 7 d of acclimation to stagnant, deoxygenated conditions, all measurements were conducted within 1–2 weeks on adventitious roots formed in these growing conditions.

Our measurements included five biological replicates for each of the aerated and stagnant treatments with each pot being the experimental unit. Each pot contained two to four plants (pseudo replicates) that served to establish a mean for each true replicate. The growth rates of wild *Oryza* species are inherently different, but for both aerated and stagnant treatments the roots grew with similar extension rates (21–29 mm d^−1^ examined for IR42 and *O. brachyantha*). Therefore, the target tissues, which were typically 30–55 mm behind the root apex, were of similar age and all target roots had been formed under the experimental conditions.

In spite of the attempt to break the strong seed dormancy of the species of wild rice by, for example, heat treatment at 50 °C for 7 d (Timple *et al.*, 2018), germination rates of *O. latifolia* and *O. granulata* remained low. Plants of these two species were thus vegetatively propagated by splitting tillers from adult plants originating from the few germinated seeds. These tillers were grown in a hydroponic solution in the same way as species with adequate germination rates (see above). Regardless of the culturing approach, all species were in their vegetative state, and we used adventitious roots (nodal roots growing from the part of the stem immersed into the nutrient solution) and none of the species formed brace roots under the given experimental conditions (see Steffens and Rasmussen (2016) for the definition of root type). Due to the rapid extension rates, there was no significant formation of fine lateral roots on the target tissue.

### Apparent permeance to O _2_

O_2_ intrusion into the root cortex was measured at contrasting external *p*O_2_ to enable calculation of the apparent permeance (*P*_A_) of the exterior cell layers to O_2_ using the approach of Peralta Ogorek *et al.* (2021). The net flux of O_2_ into the root is influenced by both tissue O_2_ consumption and resistance exerted by the diffusive boundary layer. If these parameters were known and corrected for, the permeance (permeability) coefficient could be estimated. Segments 25 mm long from 80–120 mm adventitious roots were taken 30–55 mm behind the root apex, with both cut ends sealed with lanoline to avoid longitudinal O_2_ intrusion. The root segments were gently fixed on a metal wire mesh using rubber bands and moved to a 1-litre Perspex tank. O_2_ intrusion was measured with an O_2_ microsensor (OX-25, Unisense A/S, Aarhus, Denmark) mounted on a motorized micromanipulator. A stereo microscope on a boom stand aided the positioning of the microsensor at the root surface. Using the micromanipulator, the sensor was inserted 175 μm into the root to ensure it was well inside the cortex but without reaching the stele, and then the root was immersed in DI water with a *p*O_2_ of 20.6 kPa (air equilibrium) by raising the water level in the tank. An optical microsensor (OptoMR, Unisense A/S) was positioned 2–5 mm away from the root to monitor O_2_ levels in the bulk water, and a temperature sensor (ZNTC, Unisense A/S) served to monitor temperature during the experiment. Radial O_2_ profiles have shown that the radial concentration gradient of the porous cortex is very shallow, and interspecies comparison is not challenged by differences in cortex dimension (Colmer *et al.*, 2020). O_2_ in the cortex and the bulk water close to the root were logged every 5 s using a Unisense Logger (SensorSuite v 3.1, Unisense A/S). After a minimum of 10 min of steady-state data collection, the tank was emptied without retracting the sensor from the cortex and again filled with DI water at *p*O_2_ of c. 90 kPa. The sensor was moved 5 µm further into the cortex to ensure that the self-sealing by the tissues around the sensor was intact. The signal was logged for another 10 min. The apparent permeance to O_2_ (*P*_A_; m s^−1^) was determined using the equation of Lendzian (2006):


$$\begin{array}{l} \$P_{A}=\frac{F}{A\times \Delta C}\$ \end{array}$$ (1)

where *F* (mol s^−1^) represents the intrusion rate of O_2_ via the surface area *A* (m^2^) of the root segments, and ∆*C* (mol m^−3^) is the O_2_ concentration gradient calculated as the difference in concentration between cortex and bulk water. Intrusion rate was calculated for the slope of O_2_ concentration in the cortex versus time assuming equal distribution of O_2_ in liquid phase within the root segment. For plants grown in stagnant, deoxygenated solutions, a tight ROL barrier developed in some genotypes, so cortex *p*O_2_ remained at 0 kPa. In those cases, the apparent permeance was assumed to be zero.

### Root tissue respiration

Respiration rates of root tissues were measured as O_2_ consumption by a root segment using the approach described in Winkel *et al.* (2013). Root segments of 17 mm, with the 10 mm apex region removed, were excised from adventitious roots with a total length of 80–120 mm. A root segment was placed in a 2 ml glass vial filled with medium (0.57 mM MgSO_4_, 1 mM KHCO_3_, and 0.62 mM CaCl_2_ in DI water) at air equilibrium. The tip of the O_2_ sensor (Opto-MR, Unisense A/S) was inserted through a thin capillary hole in the cap, and the decline in O_2_ concentration was measured at discrete time points. Measurements were taken at 25 °C and *p*O_2_ in the medium never approached the critical O_2_ pressure for O_2_ consumption of these root segments. During measurements, one to two blanks with DI water but without root tissue were included to account for any background O_2_ consumption by the medium. The respiration rates (nmol O_2_ s^−1^) were then calculated using the in-between rates from Unisense Rate (SensorTrace Suite v 3.2) and divided by the fresh mass (FM) of root segments to provide the final units of nmol O_2_ g^−1^ FM s^−1^. Measurements were only taken from plants in aerated nutrient solution because the presence of root barriers to radial O_2_ loss (formed in stagnant, deoxygenated solution) would impede radial O_2_ diffusion into the root and hence underestimate the respiration rate (Jiménez *et al.*, 2021a).

### Radial water loss

The barrier to ROL has recently been shown to restrict diffusion of not only O_2_ but also other gases, and therefore radial water loss (RWL) from the root segment was measured using the approach of Peralta Ogorek *et al.* (2021). Briefly, 50 mm root segments were cut from 100–120 mm adventitious roots at a distance of 30 mm behind the root apex. The diameter of each root segment was determined using a digital calliper, allowing for the calculation of root surface area. For each replicate, a minimum of 150–200 mg FM was prepared, corresponding to six to eight root segments harvested from two to four plants in the same pot. The cut ends were sealed with Vaseline and were kept wet until the start of the recordings when excess water was removed by blotting dry using paper towels. Segments were then rapidly placed on a five-digit balance (Mettler Toledo Analytical Balance ME54 with LabX direct balance 2.4 software) with silica gel in the weighing chamber to maintain low humidity of <30% (HOBO UX100-011 Temperature and RH data logger, Onset). Measurements were collected at 22–25 °C. Cumulative water loss was automatically recorded every minute over a period of 1 h after which the root segments were oven-dried at 50 °C to constant weight. Rates of radial water loss (μmol H_2_O m^−2^ s^−1^) were calculated as loss in mass between two time points divided by the surface area of the root segments. To enable comparisons among species and treatment, the point when the root samples had lost 15% of the total water content was chosen for comparing rates of RWL as this roughly represents the tissue water pool exterior to the root exodermis (Peralta Ogorek *et al.*, 2021).

### Root and root tissue dimensions

Images of root cross-sections were obtained from the same root sample used for measuring the apparent permeance to O_2_ (see above). Cross-sections were hand cut using a razor blade. Root, cortex, and stele diameters were obtained from cross-sections using a white light microscope (BX60, Olympus Optical Co. Ltd, Japan) and image processing software (cellSens Entry 3.1, Olympus) to enable calculation of the cortex-to-stele ratio. Consequently, the root anatomical traits represent the position 55–60 mm behind the root apex.

### Root tissue porosity

Root porosity (percentage gas volume per unit of root volume including both small intercellular spaces and aerenchyma) was determined using the pycnometric displacement technique and the equations described by Sojka (1988). Four to five adventitious roots (80–120 mm in length) were obtained from each pot. Segments 25 mm long were taken 30–55 mm behind the root apex. First, the weight (*W*_P_) of the pycnometer filled with DI water was measured on a five-decimal balance. Next, the fresh mass of the root segments was recorded (*W*_R_), whereafter the segments were inserted into the pycnometer and filled with DI water before weighing (*W*_B+P_). The root segment was then removed and homogenized using a mortar and pestle to eliminate any air spaces. The homogenized tissues were added back to the pycnometer, topped up with DI water, and weighed (*W*_A+P_). Finally, the root tissue porosity was calculated as


$$\begin{array}{l} \$Porosity\;\;\;\left(\;\%\;\;\;gas\text{-}f\;illed\;\;\;volume\right)=\frac{W_{A+P\;}-W_{B+P\;\;\;}}{W_{R}+W_{P\;}-W_{B+P\;}}\times 100\$ \end{array}$$ (2)

### Permeability of the root apoplast

Permeability of the root apoplast was visualized using periodic acid–Schiff’s reagent staining following the procedure of Soukup *et al.* (2002). Periodic acid oxidizes cell walls producing aldehydes that later stain purple with Schiff’s reagent. Briefly, 25 mm root segments were cut from 100–120 mm adventitious roots at a distance of 30 mm behind the root apex, sealed on the cut ends with lanoline, and incubated in 0.1% (w/v) H_5_IO_6_ for 1 h. Segments were then washed with DI water and incubated for 1 h in a reducing solution containing 2% (w/v) KI, 2% (w/v) Na_2_S_2_O_3_, and 2% (v/v) 1 M HCl followed by rinsing with DI water. Cross-sections from the middle of the root section were hand cut using a razor blade or cut by a vibrating-blade microtome (Leica VT1200S, Leica Biosystems Nussloch GmbH, Germany), stained with Schiff’s reagent for 10 min, and mounted in 70% (v/v) glycerol. Purple coloration indicating penetration of periodic acid into cell layers was visualized under a white light microscope (BX60, Olympus).

### Data analyses

#### Modelling of maximum root length

We predicted the maximum root length of the eight wild species of *Oryza* and three genotypes of *O. sativa*. We assumed that O_2_ can only be supported by internal molecular diffusion, i.e. no O_2_ source from the nutrient solution, using a modified version of the model first developed by Armstrong (1979) and subsequently applied in multiple studies (e.g. Pedersen *et al.*, 2021b). In addition to O_2_, it is well-known that sugars and amino acids can also limit root extension. In anoxic soils, phloem transport ceases but is resumed once O_2_ is re-supplied (Waters *et al.*, 1991), demonstrating that the main mechanism controlling root extension is molecular O_2_ in the apex region. The model assumes internal gas phase diffusion of O_2_ from shoot to root apex, and we considered three scenarios: (i) no root barrier to ROL, (ii) a partial barrier to ROL, and (iii) a tight barrier to ROL. The modified model we utilized was as follows:


$$\begin{array}{l} \$L=\sqrt{\frac{2C_{0}D\;\varepsilon \tau }{M_{T}S}}\$ \end{array}$$ (3)

where *L* is the maximum aerated path length equalling maximum root length, *C*_0_ is the mean O_2_ status at the root–shoot junction assumed here to be at atmospheric equilibrium (2.7 × 10^−4^ g cm^−3^), *D* is the diffusion coefficient of O_2_ (0.258 cm^2^ s^−1^, at 30 °C), ε is the fractional root porosity, τ is the tortuosity factor (assumed to be 1.0), *M*_T_ is the root tissue respiration (assumed to be constant along the root) and *S* is soil O_2_ demand (used to simulate barrier formation; 1 for tight barrier, 2–5 for partial barrier, and 6 for no barrier)—the latter approach was introduced by Pedersen *et al.* (2021b) as an addition to the original model that assumes zero ROL. We defined *S* based on the percentiles of tissue permeance to O_2_ under aerated conditions (*n*=55): 0–10% (permeance<1.52 × 10^−7^ m s^−1^), *S*=1; 10–20% (1.52 × 10^−7^<permeance<2.12 × 10^−7^), *S*=2; 20–30% (2.12 × 10^−7^<permeance<2.56 × 10^−7^), *S*=3; 30–40% (2.56 × 10^−7^<permeance<3.02 × 10^−7^), *S*=4; 40–50% (3.02 × 10^−7^<permeance<4.01 × 10^−7^), *S*=5; and 50–100% (4.01 × 10^−7^<permeance), *S*=6.

#### Phenotypic plasticity

The relative distance plasticity index (RDPI) was calculated based on key root traits (tissue porosity, root diameter, cortex diameter, stele diameter, cortex-to-stele ratio, RWL, and apparent permeance to O_2_) obtained during ‘drained’ and ‘flooded’ conditions following Valladares *et al.* (2006). The RDPI scale ranges from 0 (no plasticity) to 1 (maximum plasticity) and represents the relative phenotypic distance between individuals of the same genotype exposed to contrasting environments (Marchiori *et al.*, 2017). We constructed a matrix (2 × 5) of each trait for each species/genotype, with rows (*i*) representing the growing conditions and columns (*j*) representing the individual replicate under each growing condition. We considered two growing conditions (*i*=1, 2; aerated representing ‘drained’ and stagnant, deoxygenated representing ‘flooded’ conditions) and five individual replicates of each genotype (*j*=1, 2, 3, 4, 5).

#### Statistical analyses

GraphPad Prism 8 (v.8.0.2, GraphPad Software Inc., La Jolla, CA, USA) and SPSS Statistics (v.28.0.0.0, IBM Corp., Armonk, NY, USA) were used to prepare graphs and conduct statistical analyses. Comparison of means was conducted using either one-way ANOVA followed by Tukey’s HSD (honestly significant difference) post-hoc test or two-way ANOVA followed by Šidák’s test. To evaluate if datasets met the assumptions for parametric analysis, homoscedasticity and residual plots were used. Stele diameter and CSR were log-transformed to meet ANOVA assumptions (normality and homoscedasticity). Data on apparent permeance to O_2_ were square root transformed, which improved normality and homoscedasticity but still did not formally meet the requirements. However, ANOVA is considered very robust to unequal variances as long as the number of replicates is similar (Motulsky, 2014), so we deemed the use of two-way ANOVA on untransformed data acceptable. Correlations between apparent permeance to O_2_ and radial water loss were analysed by calculating non-parametric Spearman rank correlation coefficients because of the lack of bivariate data normality and relationships being non-linear. In all cases, we used a significance level of α=0.05, and non-transformed data are shown in the figures. The number of replicates, *P*-values, and statistical tests used are indicated in relevant figure captions.

Moreover, hierarchical clustering on principal components (HCPC) was performed on plant traits in order to group species in homogeneous clusters. HCPC was applied on the first five principal components (94.3% of explained variance), and species coordinates were used as a basis for the clustering. The agglomerative hierarchical clustering followed Ward’s criterion implemented by *k*-means aggregation to achieve the optimal partitioning. HCPC analysis was performed using the ‘FactoMineR’ package in R (version 4.0.3; Husson *et al.*, 2010).

## Results

### Differences in *Oryza* root porosity and respiration rates result in contrasting predicted maximum root lengths

**Fig. 1.**
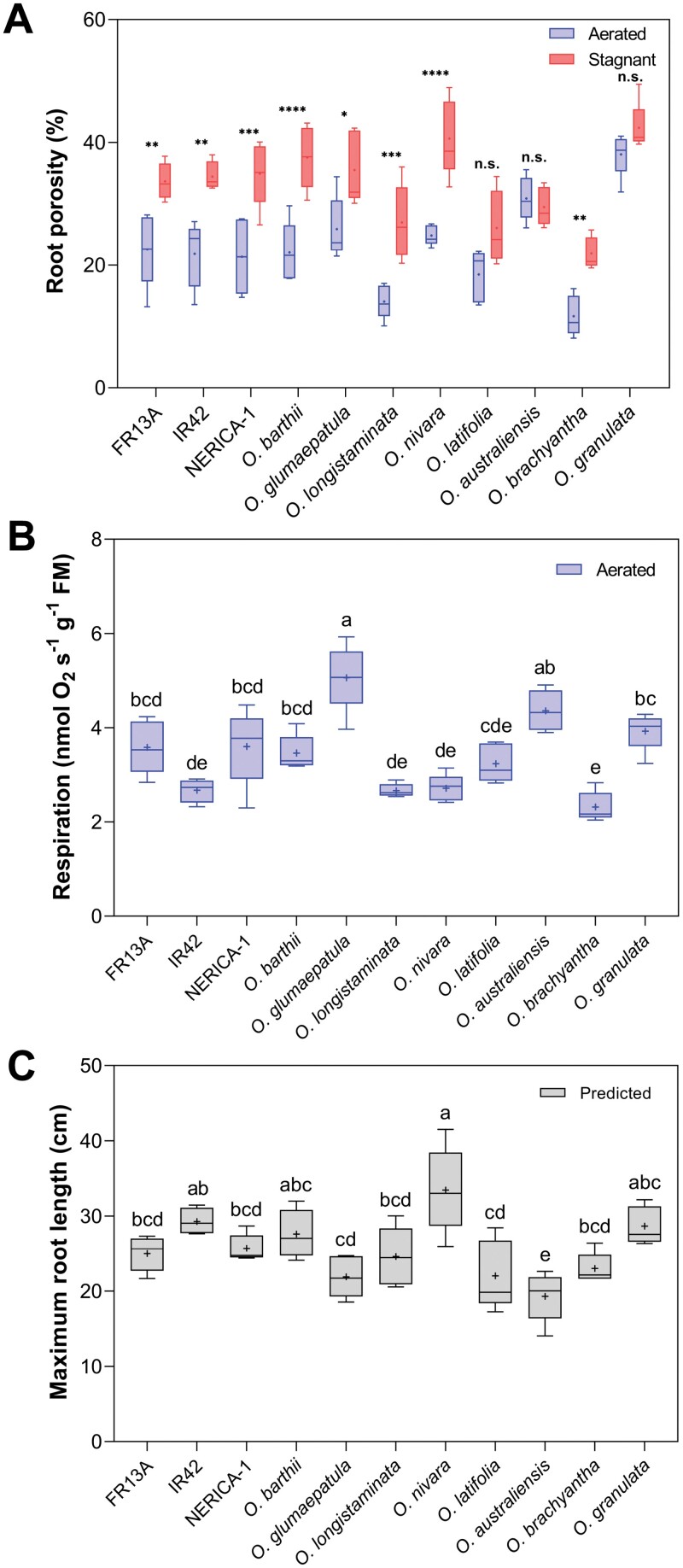
Root porosity, root respiration rates, and maximum root length of eight wild relatives of rice and three genotypes of *O. sativa* (FR13A, IR42, and NERICA-1). (A) Root porosity in aerated or stagnant, deoxygenated hydroponics was expressed as percentage gas-filled space of total root volume. Segments 25 mm long from 80–120 mm adventitious roots were taken 30–55 mm behind the root apex. Two-way ANOVA showed significant effects of growing conditions (*P*<0.0001), species (*P*<0.0001), and also growing conditions×species (*P*<0.001). Asterisks indicate significant differences (Šidák’s comparisons test): *P<0.05, **P<0.01, ***P<0.001, ****P<0.0001; ns, not significant. (B) Respiration rates of root segments excised from plants grown under aerated hydroponics. Root segments of 17 mm without the 10 mm apex were from 80–120 mm-long adventitious roots. (C) Predicted maximum root length based on root porosity, root respiration, and the presence or absence of a barrier to ROL. In (B, C), one-way ANOVA showed significant differences among means (*P*<0.0001). Different letters denote significant differences among species in Tukey’s comparisons test (α=0.05). The box and whisker plot shows the mean (+, *n*=5), the median (horizontal line), the first and third quartiles (box), and minimum and maximum (whiskers).

We aimed at characterizing key root traits of rice and its wild relatives. A main trait involved in internal aeration is root tissue porosity (Armstrong, 1979), measured in the present study using segments 30–55 mm behind the root apex. Indeed, root porosity differed significantly among species and was also influenced by growing conditions (Fig. 1A; Supplementary Fig. S1). Under aerated conditions mimicking drained soils, *O. granulata* and *O. australiensis* had the highest root porosity (38% and 31%, respectively), while *O. brachyantha* and *O. longistaminata* had the lowest root porosity (12% and 14%, respectively) (Fig. 1A). Under stagnant, deoxygenated conditions mimicking flooded soils, *O. granulata* and *O. nivara* had the highest porosity (42% and 41%, respectively), while *O. brachyantha* had the lowest (22%) (Fig. 1A). With the exception of *O. australiensis*, *O. latifolia*, and *O. granulata*, growth in stagnant, deoxygenated conditions significantly increased root porosity compared with aerated conditions, with the greatest increases of 1.6- to 1.9-fold observed in *O. nivara*, *O. barthii*, and *O. longistaminata*. (Fig. 1A). In summary, all wild species and the three genotypes of *O. sativa* possessed root porosities characteristic of wetland plants (Colmer and Voesenek, 2009) and the root porosity trait of the majority of species also showed considerable plasticity.

Another key root trait known to be influenced by growing conditions is maximum root length. However, our experimental approach was not specifically designed to obtain maximum root length and consequently we chose a modelling approach. The modelling required root tissue respiration as an input parameter, and therefore we determined O_2_ consumption by root segments grown in an aerated condition. Root respiration rates varied significantly among species with the highest (*O. glumaepatula*, 5.1 nmol O_2_ s^−1^ g^−1^ FM) being more than twice that of the lowest (*O. brachyantha*, 2.3 nmol O_2_ s^−1^ g^−1^ FM (Fig. 1B). The range in root respiration in combination with variation in root tissue porosity was clearly reflected by the root length model. Predicted maximum length of adventitious roots under stagnant, deoxygenated conditions varied between 19.3 cm in *O. australiensis* and 33.5 cm in *O. nivara* (Fig. 1C). These findings show that even if the root length model is relatively simple, it serves as a useful tool to predict maximum root length in a selection of rice species.

### Growth under stagnant, deoxygenated conditions induced a ROL barrier in most species

**Fig. 2.**
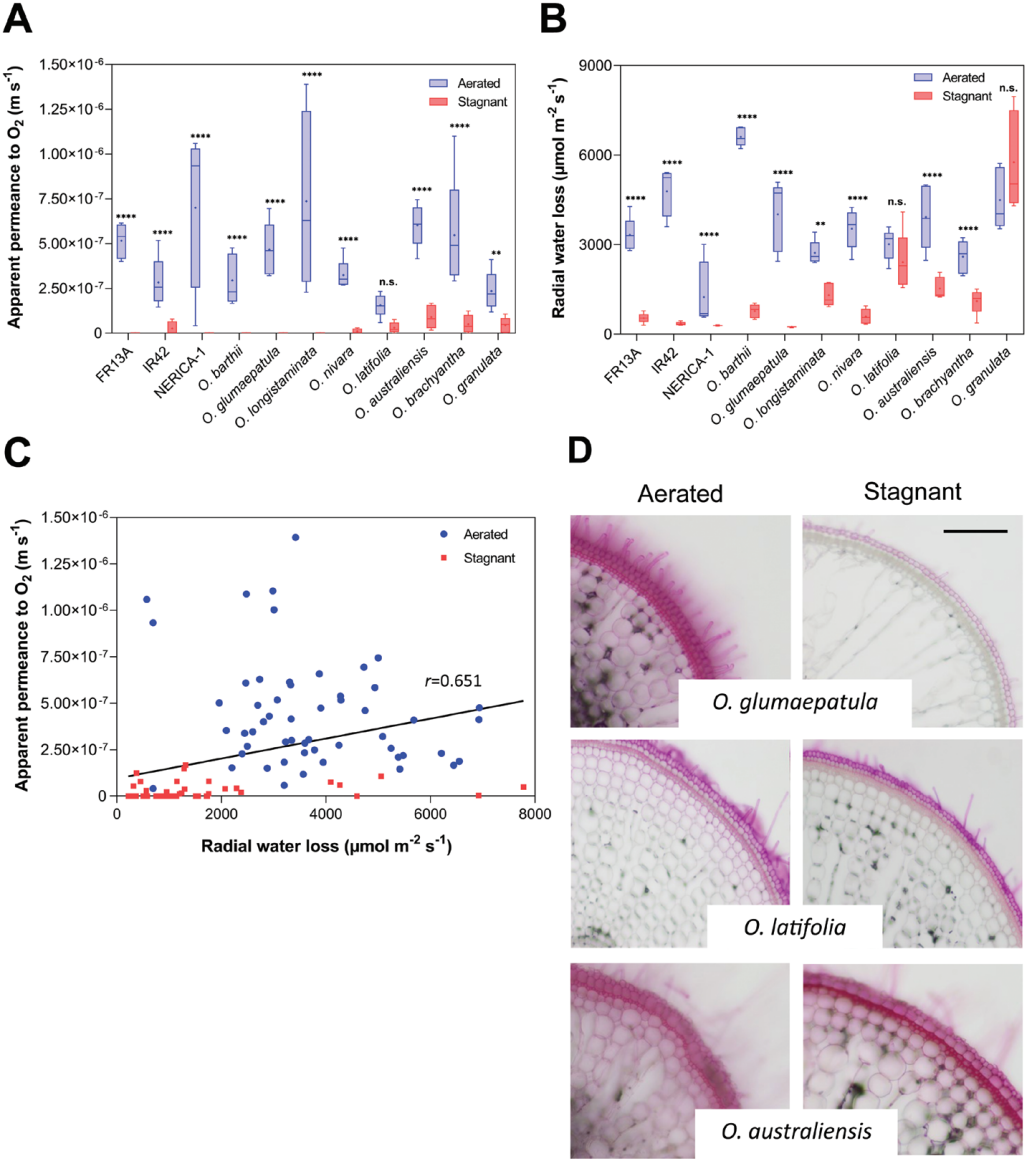
Apparent permeance to O_2_, radial water loss (RWL), stained root cross-sections, and relationship between apparent permeance to O_2_ and RWL in eight wild relatives of rice and three genotypes of *O. sativa* (FR13A, IR42, and NERICA-1). (A) Apparent permeance to O_2_ of *Oryza* species grown in aerated or stagnant, deoxygenated nutrient solution. The apparent permeance to O_2_ of the outer part of the root was measured on 25 mm-long segments from 80–120 mm-long adventitious roots using an O_2_ microsensor positioned inside the root cortex. Two-way ANOVA showed significant effects of growing conditions (*P*<0.0001), species (*P*=0.0015), and also growing conditions×species (*P*=0.0011). (B) Rates of RWL from roots of *Oryza* species grown in aerated or stagnant, deoxygenated nutrient solution. RWL was determined as the first derivative from curves of cumulative water loss at 85 % water content. Two-way ANOVA showed significant effects of growing conditions (*P*<0.0001), species (*P*<0.0001), and also growing conditions×species (*P*<0.0001). (C) Relationship between apparent permeance to O_2_ (m s^−1^) and RWL (μmol H_2_O m^−2^ s^−1^). Spearman correlation, *P*<0.0001, *r*=0.651, *n*=109. In (A, B) the box and whisker plot shows the mean (+, *n*=5), the median (horizontal line), the first and third quartiles (box), and minimum and maximum (whiskers). Asterisks indicate significant differences (Šidák’s comparisons test). **P<0.01; ***P<0.001; ****P<0.0001; ns, not significant. (D) Purple apoplastic tracer applied to root sections and cross-sections taken from position 40–45 mm behind the root apex of 100–120 mm-long adventitious roots. To facilitate comparison, *O. glumaepatula*, *O. latifolia*, and *O. australiensis* are also shown in Supplementary Fig. S2 along with the remaining species and genotypes. Scale bar: 200 µm.

The root barrier to ROL is considered one of the most important root traits enabling growth in flooded soils (Colmer, 2003b). We used different approaches to quantify radial resistance to gas diffusion and visualize radial resistance to ion diffusion across the outer root cell layers. Apparent permeance (*P*_A_) to O_2_ was calculated to enable a quantitative analysis of the diffusive resistance to O_2_ of the cell layers exterior to the cortex and the influence of a possible ROL barrier. *P*_A_ to O_2_ was influenced significantly by growing conditions but responses varied among species (Fig. 2A; Supplementary Fig. S1). Under aerated conditions, roots had higher apparent permeance, i.e. radial resistance to O_2_ diffusion was low. *Oryza longistaminata* had the highest apparent permeance (7.37 × 10^−7^ m s^−1^), while *O. latifolia* had the lowest (1.56 × 10^−7^ m s^−1^) (Fig. 2A). In all but one species (*O. latifolia*), stagnant, deoxygenated conditions led to a substantial decline in *P*_A_ to O_2_ and apparent permeance was below detection limit in three of the wild species and in two genotypes of *O. sativa* (Fig. 2A). These results clearly show that stagnant, deoxygenated conditions induce a strong resistance to O_2_ diffusion in the outer root cell layers.

Radial water loss (RWL) from root segment has recently been shown to correlate positively with ROL in rice (Peralta Ogorek *et al.*, 2021), and therefore we also quantified RWL in the eight wild relatives of rice and the three genotypes of *O. sativa*. Similarly to *P*_A_ to O_2_, RWL was influenced significantly by growing conditions but responses also differed among species (Fig. 2B; Supplementary Fig. S1). Under aerated conditions, RWL ranged from 6612 µmol H_2_O m^−2^ s^−1^ in *O. barthii* to only 1242 µmol H_2_O m^−2^ s^−1^ in the genotype ‘NERICA-1’ (Fig. 2B). For most species, growth in stagnant, deoxygenated conditions significantly reduced RWL (Fig. 2B) following the pattern of *P*_A_ to O_2_. Interestingly for *O. latifolia*, neither *P*_A_ to O_2_ nor RWL differed between aerated or stagnant, deoxygenated conditions. The most dramatic response to growing conditions was observed in *O. glumaepatula* where RWL was reduced by 94% under stagnant, deoxygenated conditions compared with aerated conditions. Our multispecies approach confirmed the previous observation that RWL and ROL (measured as *P*_A_ to O_2_) are interrelated. Indeed, we found a strongly significant positive correlation between apparent permeance to O_2_ and RWL (Fig. 2C), further supporting the recent observation that the barrier to ROL is also restricting RWL from roots of rice (Peralta Ogorek *et al.*, 2021).

The potential presence of an apoplastic barrier to ion diffusion in the exodermis was visualized using periodic acid as tracer. The responses of the exodermis to changes in growing conditions can be divided into three types as follows. (i) Neither aerated nor stagnant, deoxygenated conditions resulted in formation of an apoplastic barrier in the root exodermis of *O. australiensis*, *O. nivara* and *O. granulata*. (ii) In both aerated and stagnant, deoxygenated conditions, a partial barrier was formed so that the cortex was weakly stained regardless of growing conditions, which was the case for *O. latifolia*. (iii) In aerated conditions, the apoplast of the exodermis was permeable and the cortex stained purple before the tracer was blocked by the apoplastic barrier of the endodermis, while in stark contrast, stagnant, deoxygenated conditions resulted in a tight apoplastic barrier in the exodermis and only the outermost cell layers were stained—this scenario applied for *O. longistaminata*, *O. barthii*, *O. glumaepatula*, *O. brachyantha*, and the three genotypes of *O. sativa* (Fig. 2D; Supplementary Fig. S2). Interestingly, the species included in type (i) are mostly species with high *P*_A_ to O_2_ regardless of growing conditions, whereas type (ii) shows no apparent relationship with *P*_A_ to O_2_, and the type (iii) response shows large overlap with species having *P*_A_ to O_2_ below detection limit (Fig. 2A). This indicates that roots harbouring a strong apoplastic barrier also had features in the exodermis that restricted ROL.

### Soil flooding increased root thickness in all *Oryza* species

**Fig. 3.**
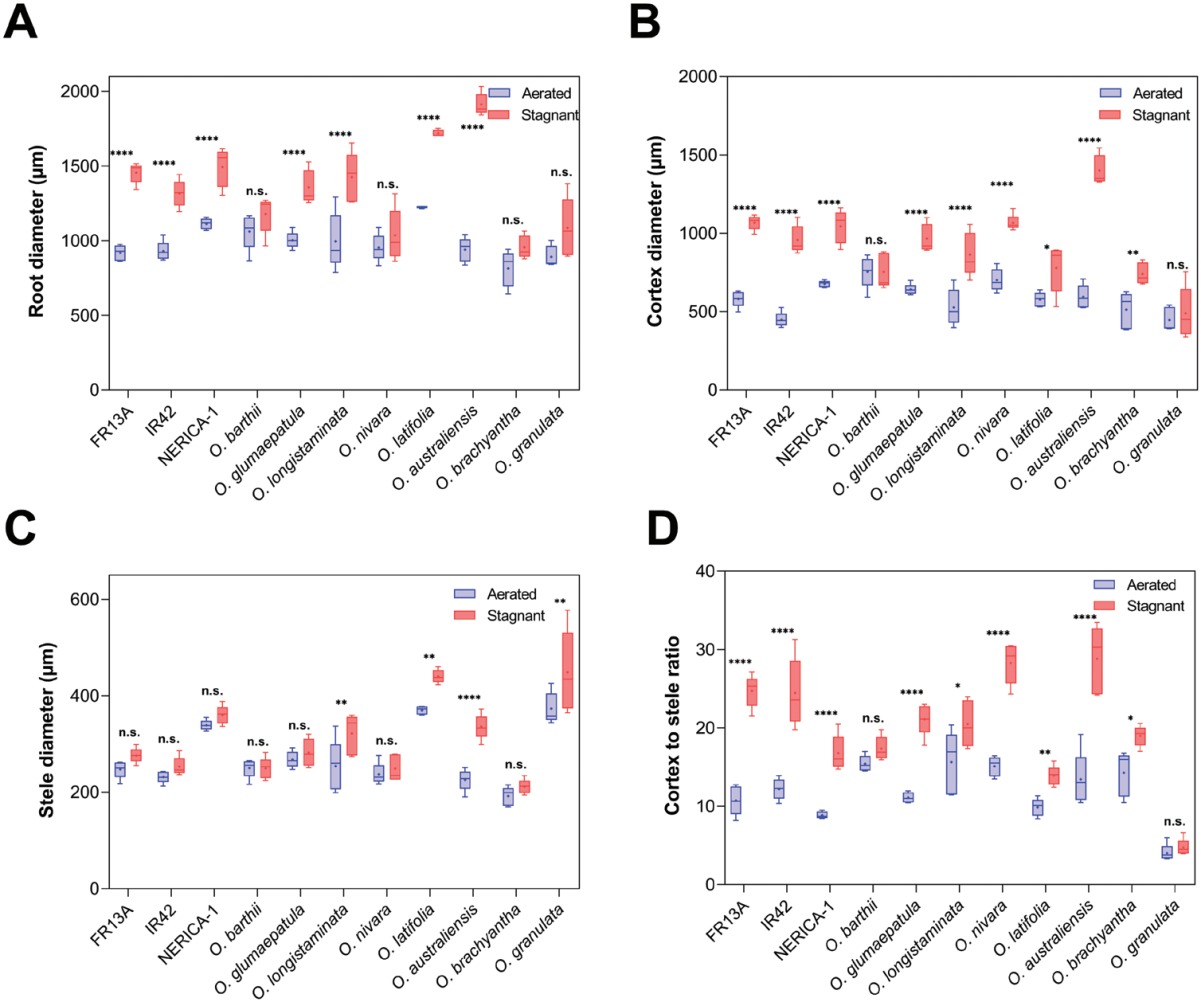
Root and root tissue dimensions of eight wild relatives of rice and three genotypes of *O. sativa* (FR13A, IR42, and NERICA-1) in aerated or stagnant, deoxygenated nutrient solution. Root diameter (A), cortex diameter (B), stele diameter (C), and cortex-to-stele ratio (D) of 80–120 mm-long adventitious roots with measurements taken 55–60 mm behind the root apex. Two-way ANOVA showed significant effects of growing conditions (*P*<0.0001), species (*P*<0.0001), and also growing conditions×species (*P*<0.0001) in (A–D). The box and whisker plot shows the mean (+) of five true replicates, the median (horizontal line), the first and third quartiles (box), and minimum and maximum (whiskers). Asterisks indicate significant differences (Šidák’s comparisons test): *P<0.05, **P<0.01, ****P<0.0001; ns, not significant.

It is generally accepted that thick roots and a high cortex-to-stele ratio (CSR) enhance tissue O_2_ status under flooded conditions (Armstrong and Beckett, 1987; McDonald *et al.*, 2002; Garthwaite *et al.*, 2003; Yamauchi *et al.*, 2014). Indeed, root, cortex, and stele dimensions were all influenced by growing conditions and species (Fig. 3A–C; Supplementary Fig. S1). In fact, *O. australiensis* increased root thickness 2.0-fold in stagnant, deoxygenated conditions. In the majority of species, stagnant, deoxygenated conditions resulted in a significant increase in root and cortex thickness, whereas the stele in most cases did not respond significantly to growing conditions (Fig. 3A, C). Only the stele diameter of *O. longistaminata* and *O. australiensis* increased significantly under stagnant, deoxygenated conditions compared with aerated conditions (Fig. 3B).

A large CSR enhances the capacity for O_2_ transport along roots because diffusion of O_2_ to the root apex takes place via the porous cortex (Pedersen *et al.*, 2021b). Under aerated conditions, *O. longistaminata* and *O. barthii* had the highest CSR (15.6 and 15.4, respectively), while *O. granulata* had the lowest (4.0) (Fig. 3D). Under stagnant, deoxygenated conditions, *O. australiensis* had the highest CSR (28.8), while CSR of *O. granulata* was still the lowest (4.7) (Fig. 3D). CSR as a root trait can respond substantially to changes in the environment consistent with the fact that CSR of three genotypes more than doubled under stagnant, deoxygenated compared with aerated conditions. In the majority of species, stele thickness did not respond to growing conditions, and therefore the increase in CSR was mainly caused by an increase in cortex dimension rather than a decrease in stele thickness.

### *Oryza sativa* harbours root phenotypic plasticity for soil flooding tolerance comparable to its wild relatives

**Fig. 4.**
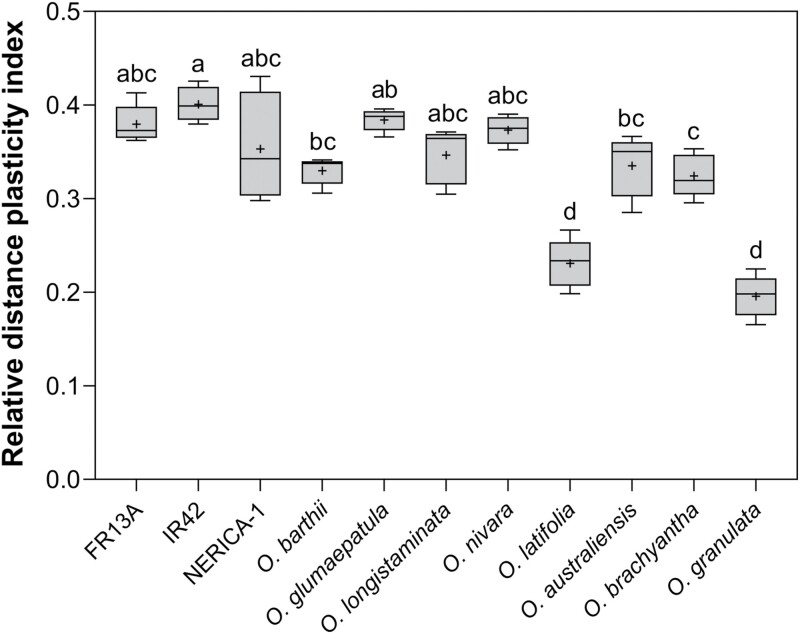
Mean relative distance plasticity index of eight wild relatives of rice and three genotypes of *O. sativa* (FR13A, IR42, and NERICA-1) subjected to contrasting growing conditions (aerated or stagnant, deoxygenated nutrient solution). The index is based on the mean responses of seven key root traits: RWL, apparent permeance (*P*_A_) to O_2_, root porosity, cortex-to-stele ratio, root diameter, cortex diameter, and stele diameter. The box and whisker plot shows the mean (+) of five true replicates, the median (horizontal line), the first and third quartiles (box), and minimum and maximum (whiskers). One-way ANOVA showed significant differences among means (*P*<0.0001). Different letters denote significant differences among species in Tukey’s comparisons test (α=0.05).

All of the above root traits responded to growing conditions, but the magnitude of responses varied among the eight wild species of rice and the three genotypes of *O. sativa*. We therefore constructed a relative distance plasticity index (RDPI) to quantify phenotypic plasticity, thereby providing a single number expressing the capacity of each species to respond to growing conditions. Our index includes seven key root traits: root, cortex and stele diameter, CSR, root porosity, RWL, and *P*_A_ to O_2_, and the RDPI revealed significant differences in phenotypic plasticity. The most responsive species were *O. glumaepatula* (0.38) and ‘IR42’ (0.40) both with a numerical response almost 2-fold that of *O. granulata* (0.20) (Fig. 4). Trait-specific RDPI showed large numerical variation; for example, *P*_A_ to O_2_ showed extreme plasticity with values up to 1.00 and it never declined below 0.68, whereas RDPI of the stele diameter ranged from 0.03 (hardly any response to growing conditions) to 0.20 (indicating moderate plasticity) (Supplementary Fig. S1). Surprisingly, the plasticity of key root traits observed in *O. sativa* genotypes was not lower than in any of the eight wild relatives of rice, indicating that the investigated *O. sativa* genotypes are well suited for growth in wet habitats.

### Clustering of species based on trait responses to growing conditions

**Fig. 5.**
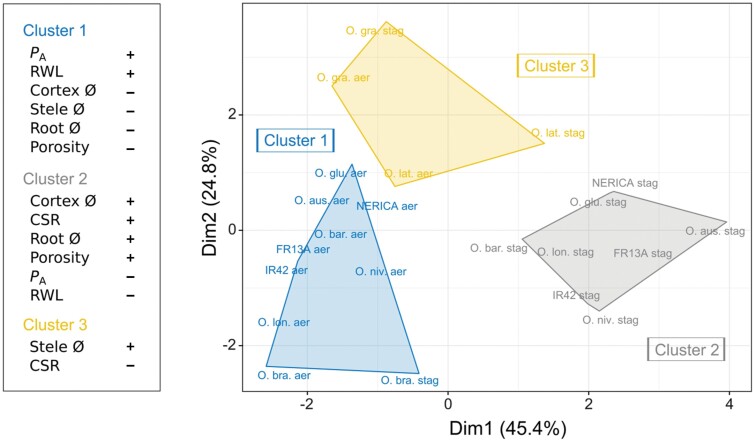
Hierarchical clustering on principal components (HCPC) was applied to key root traits. The root traits included in the analysis were permeance to O_2_ (*P*_A_), radial water loss (RWL), cortex-to-stele ratio (CSR), root, stele and cortex dimensions (Ø), and tissue porosity. Only the first two principal components are reported (70.2% of explained variance). Labels refer to the name of the species or genotype followed by the growing conditions. Species abbreviations are: FR13A, *O. sativa* ‘FR13A’; IR42, *O. sativa* ‘IR42’; NERICA, *O. glaberrima* × *sativa* ‘NERICA-1’; O. bar., *O. barthii*; O. glu., *O. glumaepatula*; O. long., *O. longistaminata*; O. niv., *O. nivara*; O. lat., *O. latifolia*; O. aus, *O. australiensis*; O. bra., *O. brachyantha*; O. gra., *O. granulata*. Growing conditions: Aer, aerated; Sta, stagnant, deoxygenated. + or − indicates traits significantly higher or lower of the overall mean of each individual trait; only statistically significant traits are shown.

Hierarchical clustering on principal components (HCPC) analysis grouped species into three well-distinguished clusters and captured a total of 70.2% of the variation on the two axes (Fig. 5). Cluster 1 primarily contains species or genotypes grown in aerated conditions whereas cluster 2 contains species grown under stagnant, deoxygenated conditions. The separation into two statistically different groups demonstrates the strength of the abiotic stress imposed by stagnant, deoxygenated conditions on all but three species of rice (*O. granulata*, *O. latifolia*, and *O. brachyantha*). *Oryza brachyantha* was assigned to cluster 1 regardless of growing conditions, i.e. it showed little response to stagnant, deoxygenated conditions and had very low values of tissue porosity (Figs 1A, 5). Six traits contributed significantly to grouping of cluster 1, and this group is characterized by high RWL and high *P*_A_ to O_2_ but low root porosity, and small stele, cortex and root diameter; these are all characteristic traits of drained conditions where a root ROL barrier is rarely formed and constitutive aerenchyma formation is limited. In contrast, cluster 2 is characterized by high root porosity, large root and cortex diameter, and high CSR, but low values of RWL and *P*_A_ to O_2_; also here the multivariate approach successfully captured traits characteristic of growth in stagnant, deoxygenated nutrient solution. Finally, cluster 3 contains only two species (*O. granulata* and *O. latifolia*) but these are grouped into this cluster irrespective of growing conditions. This group is characterized by a large stele diameter and a low CSR (Fig. 5).

## Discussion

We aimed at assessing phenotypic plasticity in key root traits associated with tolerance to soil flooding using eight wild species of rice and three genotypes of *O. sativa*. The relative distance plasticity index (RDPI) quantified phenotypic plasticity of seven root traits when exposed to contrasting growing conditions (aerated hydroponics to mimic drained soil *versus* stagnant, deoxygenated hydroponics to mimic flooded soil). Using hierarchical clustering of principal components (HCPC), we found that these growing conditions separated species into well-defined clusters based on responses in anatomical and biophysical traits. Below, we discuss the observed plasticity in root traits in the studied *Oryza* species in the context of existing knowledge and application in strategies to improve resilience to abiotic stress in cultivated rice.

### Phenotypic plasticity in key root traits varied substantially between species of wild rice

Phenotypic plasticity in root traits has been proposed as a tool to improve tolerance to abiotic stress. Interestingly, phenotypic plasticity in key root traits relevant to soil flooding of the *O. sativa* genotypes and most of the included wild species was similar, showing that the wetland traits of wild rice have generally been conserved in the three genotypes of *O. sativa*. Interestingly, studies have revealed that mechanistic responses to abiotic stress can differ in wild species and cultivated rice. Submergence tolerance of *O. grandiglumis* (Okishio *et al.*, 2014), *O. rhizomatis*, and *O. eichingeri* (Niroula *et al.*, 2012) was not conferred by *SUB1A* or *SNORKEL1*/*SNORKEL2* genes, clearly showing that flood tolerance in these species differs from our current understanding based on the *O. sativa* genotypes of FR13A (Xu *et al.*, 2006) and deepwater rice (Hattori *et al.*, 2009). Moreover, the present study included only one accession of each wild species, although there is high intra-species variation in traits related to soil flooding. In the Amazonian wetland species *O. glumaepatula*, a constitutive barrier to ROL was identified in only one of three accessions investigated (Ejiri *et al.*, 2020). We therefore propose to use georeferenced data for each available accession in combination with high-resolution GIS maps with information on soil flooding status to identify promising candidate accessions prior to laborious screening studies.

Dryland *O. granulata* stood out as a species with very low overall plasticity. The RDPI was significantly lower than for all other species except *O. latifolia*, and its low phenotypic plasticity was mainly driven by weak responses in root porosity, CSR, and *P*_A_ (Fig. 4; Supplementary Fig. S1). Our HCPC analysis also revealed this lack of response to contrasting growing conditions, where *O. granulata* and *O. latifolia* clustered separately from all the other species. Two root traits characterized cluster 3, a low CSR and a large stele, both of which are characteristic traits of plants growing in low soil moisture (Yamauchi *et al.*, 2021). Another interesting exception from the general pattern is that *O. brachyantha* clustered together with most of the aerated plants (Fig. 5). This wetland species showed unexpectedly low tissue porosity in both aerated and stagnant, deoxygenated nutrient solution. Phenotypic plasticity of root traits has previously been linked to habitat type. Across 91 plant species, inducible root porosity remained low in non-wetland plants regardless of growing conditions, but increased as a response to soil flooding in wetland plants (Justin and Armstrong, 1987). Similarly, adventitious root formation by plants as a response to soil flooding, as well as the adaptive characteristics of these roots, was positively correlated to the frequency of soil flooding in their natural habitats (Visser *et al.*, 1996). In fact, *O. granulata* showing the lowest phenotypic plasticity is characterized as a dryland species not found in standing water (Vaughan, 1994; Shi *et al.*, 2020). Its inability to respond to soil flooding is therefore likely of little competitive importance in its natural habitat.

### Root porosity generally showed high phenotypic plasticity but not in all wild species

Rice constitutively forms lysigenous aerenchyma leading to moderate tissue porosity in the cortex. Furthermore, soil flooding induces aerenchyma, and therefore also tissue porosity, as cortical cell death is initiated by accumulation of the gaseous phytohormone ethylene (Justin and Armstrong, 1991; Yamauchi and Nakazono, 2022). In accordance, root porosity in the three *O. sativa* genotypes increased significantly from 21–23% gas-filled spaces in aerated nutrient solution to 34–35% in stagnant, deoxygenated conditions (Fig. 1A). However, root porosity is not uniform along the root axis; with very low porosity in the expanding zone of the root increasing with distance from the root apex (e.g. Kotula *et al.*, 2009). Our trait measurements occurred 30–55 mm behind the root apex, representing where the barrier to ROL is typically induced in rice (Colmer, 2003a). In this position, the observed percentage in root porosity, as well as the magnitude in response to growing conditions, is similar to those reported for an upland rice cultivar (‘Azucena’; Kotula *et al.*, 2009) and a lowland rice (‘Nipponbare’; Yamauchi *et al.*, 2019). Since formation of cortical aerenchyma is partly controlled by tissue age (Yamauchi *et al.*, 2020), directly comparing root tissue porosity between species is challenging even within the same study, unless tissue age can be accurately determined from root growth rates.

Nevertheless, root extension rates were not determined in all species and therefore species-to-species as well as intra-species direct comparisons should be treated with caution. However, root tissue porosity changed substantially in *O. longistaminata*, *O. barthii*, *O. glumaepatula*, *O. nivara*, and *O. brachyantha*, but not in *O. latifolia*, *O. granulata*, and *O. australiensis* (Fig. 1A). Species with high constitutive porosity show less potential to further increase porosity under soil flooding (Justin and Armstrong, 1987). Thus root porosity in *O. granulata* exceeded 40% under aerated conditions, and did not increase further under stagnant, deoxygenated conditions (Fig. 1A). Furthermore, an *O. glumaepatula* accession had very high constitutive aerenchyma formation, and a subsequent lack of inducible aerenchyma when exposed to stagnant, deoxygenated conditions (Ejiri *et al.*, 2020). In contrast, *O. sativa* normally shows high phenotypic plasticity in this trait (e.g. Colmer, 2003a). The eco-physiological benefits from inducible aerenchyma are evident since a large cross-sectional gas-filled area in the cortex represents a low resistance pathway for gas phase diffusion of O_2_ from shoot to root apex (Colmer, 2003b). However if the root porosity is constitutively high, a further increase may compromise root function by affecting water uptake or structural integrity (Lynch *et al.*, 2021). Unexpectedly, root porosity of the wetland species *O. brachyantha* was 12% and 22% in aerated and stagnant, deoxygenated conditions, respectively, with low constitutive aerenchyma and low inducible aerenchyma (Table 1). Even though this trait was measured only 30–55 mm behind the root apex, aerenchyma is normally pronounced in this position and should also respond to stagnant, deoxygenated conditions (Kotula *et al.*, 2009; Yamauchi *et al.*, 2019). Consequently, we cannot rule out that more pronounced changes in cortical porosity occur further back in the root although this would be of little functional importance for the O_2_ supply to the growing root apex. Interestingly, this species forms numerous but rather short (max. 200 mm) roots when growing in stagnant, deoxygenated conditions, showing the disadvantages of low cortical porosity in anoxic soils.

### Growing conditions influence apparent permeance to O _2_

By restricting diffusional loss to anoxic soils, the root barrier to ROL is a key root trait that confers tolerance to soil flooding by greatly improving root tissue O_2_ status (Colmer, 2003b). The barrier to ROL is inducible in genotypes of *O. sativa* (e.g. Colmer, 2003a) but is constitutively formed in the most basal part of the root (60–100 mm behind the root apex) in three *O. glumaepatula* accessions (Ejiri *et al.*, 2020). Although we measured ROL barriers and apoplastic barriers in the exodermis 30–55 mm behind the root apex, where inducible barriers in *O. sativa* genotypes normally form (Colmer, 2003a), none of the wild species formed a constitutive barrier to ROL. Regions further behind the root apex were not examined, so we cannot determine if some species form a constitutive barrier close to the root–shoot junction as in *O. glumaepatula* (Ejiri *et al.*, 2020). Instead, all but one species responded to stagnant, deoxygenated conditions by a significant reduction in apparent permeance (*P*_A_) to O_2_ (Fig. 2A), which the present study used as a diagnostic tool for ROL barrier formation.

*P*_A_ was recently introduced as a quantitative approach to characterize diffusional resistance to O_2_ and other gases by the outer part of the root (Peralta Ogorek *et al.*, 2021). Furthermore, this approach is non-destructive as measurements only require a short root segment (see Jiménez *et al.* (2021c) for evaluations of methods on ROL in roots). Root segments have also been used to characterize resistance to O_2_ diffusion across the outer part of roots using a perfusion technique, with permeability coefficients (1.3–2.0 × 10^−6^ m s^−1^ in the position 30–50 mm behind the root apex) of rice roots grown in aerated nutrient solutions indicating no formation of a barrier to ROL (Kotula and Steudle, 2009). These permeability coefficients were 2- to 10-fold higher than the *P*_A_ in the present study (Fig. 2A). Since the *P*_A_ approach in addition to the biophysical resistance to O_2_ diffusion imposed by suberization of the root exodermis also includes the component of O_2_ consumption by the outer part of the root, a lower *P*_A_ compared with the actual permeability coefficient would be expected. The significant decline in *P*_A_ as a response to stagnant, deoxygenated conditions underlined the barrier to ROL is phenotypically plastic. In 5 of 11 species/genotypes, *P*_A_ declined to below the detection level with the remaining six species/genotypes showing *P*_A_ of 1.05–9.02 × 10^−8^ m s^−1^. Without considering the species where *P*_A_ was below detection limit in stagnant, deoxygenated conditions, *P*_A_ under aerated conditions were 5- to 31-fold higher. Similarly in *Hordeum marinum*, the inducible barrier reduced ROL by 96% (Garthwaite *et al.*, 2008). Such high resistance to radial O_2_ diffusion by the suberized exodermis underlines its remarkable capacity to retain O_2_ inside the root when rice is growing in anoxic soils.

In addition to reducing O_2_ loss, the root barrier to ROL in rice also restricts radial diffusion of H_2_ and H_2_O (Peralta Ogorek *et al.*, 2021). Similarly, our measurements of radial water loss (RWL) showed that the ROL barrier significantly delays root desiccation in all but two species (Fig. 2B). However, the ROL barrier does not seem to affect root hydraulic conductivity as the dryland rice cultivar ‘Azucena’ (Ranathunge *et al.*, 2011) and *Hordeum marinum* (Garthwaite *et al.*, 2003) showed no decline in hydraulic conductivity when adventitious roots formed in aerated versus stagnant, deoxygenated nutrient solutions were compared. Since the barrier to ROL typically forms 2–3 cm behind the root apex (Colmer, 2003a) and only one of two types of lateral roots forms a ­barrier to ROL (Noorrohmah *et al.*, 2020), large proportions of the root system remain unaffected by the apoplastic barrier in the root exodermis formed in the basal part of the root. Nevertheless, the exodermal apoplastic barrier is suggested to offer some protection from desiccation in dry soil (e.g. Cruz *et al.*, 1992; Enstone *et al.*, 2002; Toulotte *et al.*, 2022).

### Predicting maximum root length from root porosity, ROL barrier strength, and rates of respiration

Due to the anoxic properties of flooded soils (Ponnamperuma, 1972), O_2_ for root extension is provided by longitudinal molecular diffusion via the porous cortex (Armstrong, 1979). Therefore, maximum root length can be predicted from the aerated path length inside roots (Armstrong, 1979). In addition to root porosity, the diffusive O_2_ transport along the root also depends on tissue O_2_ consumption (respiration) and radial O_2_ loss to the anoxic soil (Pedersen *et al.*, 2021b). Therefore, we used data on root porosity, apparent permeance (*P*_A_) to O_2_, and root respiration rates to predict maximum root length (Eq. 3). Except for two of the wild species (*O. australiensis* and *O. nivara*), the range in modelled maximum root lengths in this study (19.3–33.5 cm) was similar to that of 12 *O. sativa* genotypes grown in stagnant, deoxygenated nutrient solution (21.7–31.4 cm) (Colmer, 2003a). Only *O. nivara* attained longer predicted root lengths than some of the included *O. sativa* genotypes. *Oryza australiensis* was predicted to obtain the shortest maximum root length as it had very low root porosity, whereas predicted maximum root length of *O. granulata* was similar to the *O. sativa* genotypes. The model to predict maximum root length, based on the three variables listed above, accurately predicts root length (Pedersen *et al.*, 2021a). Nevertheless, growing plants in large pots in real soils is required to obtain data on maximum root length that reflects natural soil flooding conditions.

In conclusion, growth under stagnant, deoxygenated conditions altered key root traits of all included *Oryza* species and genotypes of *O. sativa*. The three genotypes of *O. sativa* all had higher phenotypic plasticity in traits conferring tolerance to soil flooding than all the wild species, possibly demonstrating genetic adaptation of these species to soil flooding such that alleles coding for broader plasticity have been lost (Brooker *et al.*, 2022). Only the dryland wild rice relative *O. granulata* had significantly lower phenotypic plasticity in the considered root traits. Divergent growing conditions did not result in statistically significant separation of trait responses in *O. granulata* and *O. latifolia*. Although none of the wild species showed superior root plasticity to the *O. sativa* genotypes, our results support implementing a targeted approach to sourcing wild rice germplasm for abiotic stress tolerance. Such an approach should be based on geo-referenced species distribution maps in combination with climatic data and GIS analysis (Atwell *et al.*, 2014). That relatives of wild rice harbour novel traits conferring tolerance toward complete submergence (Okishio *et al.*, 2014) and form a constitutive ROL barrier (Ejiri *et al.*, 2020), combined with novel insights into salt tolerance mechanisms in the salt marsh species, *Oryza coarctata* (Parmar *et al.*, 2020), further supports such an approach.

## Supplementary data

The following supplementary data are available at *JXB* online.

Fig. S1. Trait-specific relative distance plasticity index of eight wild relatives of rice and three genotypes of *O. sativa*.

Fig. S2. Stained root cross-sections of eight wild relatives of rice and three genotypes of *O. sativa*.

erad014_suppl_Supplementary_Material

## Data Availability

The data supporting the findings of this study are available from the corresponding authors, OP or MH, upon request.
