# Supplementary material for: Responses of key root traits in the genus Oryza to soil flooding mimicked by stagnant, deoxygenated nutrient solution
Source: J Exp Bot. 2023 Jan 11;74(6):2112–26. doi: 10.1093/jxb/erad014 (PMC10049916; doi:10.1093/jxb/erad014)
Supplement: erad014_suppl_Supplementary_Material [file erad014_suppl_supplementary_material.pdf]

SUPPLEMENTARY DATA for "Responses of key root traits in the genus *Oryza* to soil flooding"

Shuai Tong[1,a], Johan Emil Kjær[1,a], Lucas León Peralta Ogorek[1], Elisa Pellegrini[1,2], Zhiwei Song[1], Ole Pedersen[1,3,*] and Max Herzog[1,*]

[1] Department of Biology, University of Copenhagen, Universitetsparken 4, 3rd floor, 2100 Copenhagen, DENMARK

[2] Department of Agricultural, Food, Environmental and Animal Sciences, University of Udine, Via delle Scienze 206, 33100 Udine, ITALY

[3] School of Agriculture and Environment, The University of Western Australia, 35 Stirling Highway, WA 6009, Australia

[a] these authors contributed equally to this work

[*] corresponding author: opedersen@bio.ku.dk and herzogmax@bio.ku.dk

Supporting figures

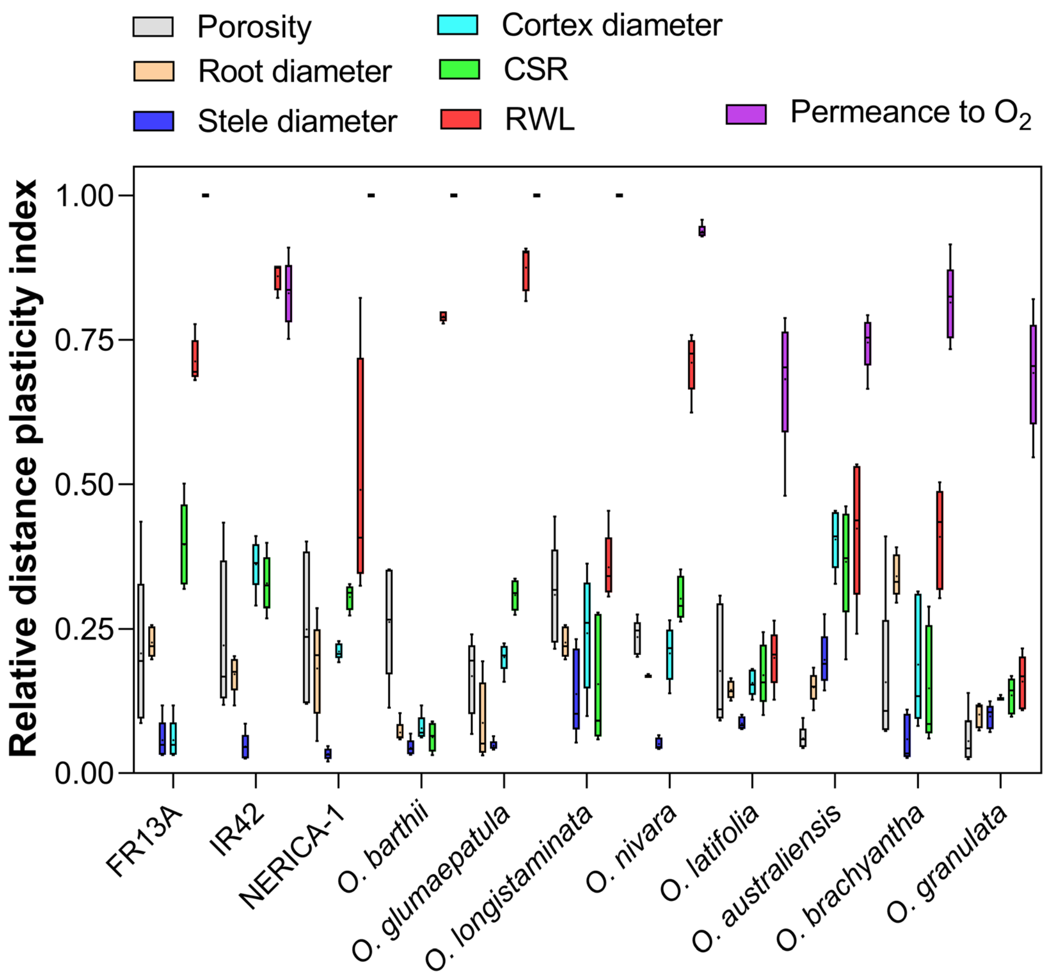

<u>Fig. S1.</u>

Trait-specific relative distance plasticity index of 8 wild relatives of rice and 3 genotypes of *O. sativa* (FR13A, IR42 and NERICA-1) subjected to contrasting growth conditions varying (aerated or stagnant, deoxygenated nutrient solution). The box-whisker plot shows the mean (+) of five true replicates, the median (horizontal line) and minimum or maximum (whiskers). One-Way ANOVA showed significant differences among means ($P < 0.0001$). The mean RDPI shown in Fig. 4 is based on these individual trait indices.

Fig. S2

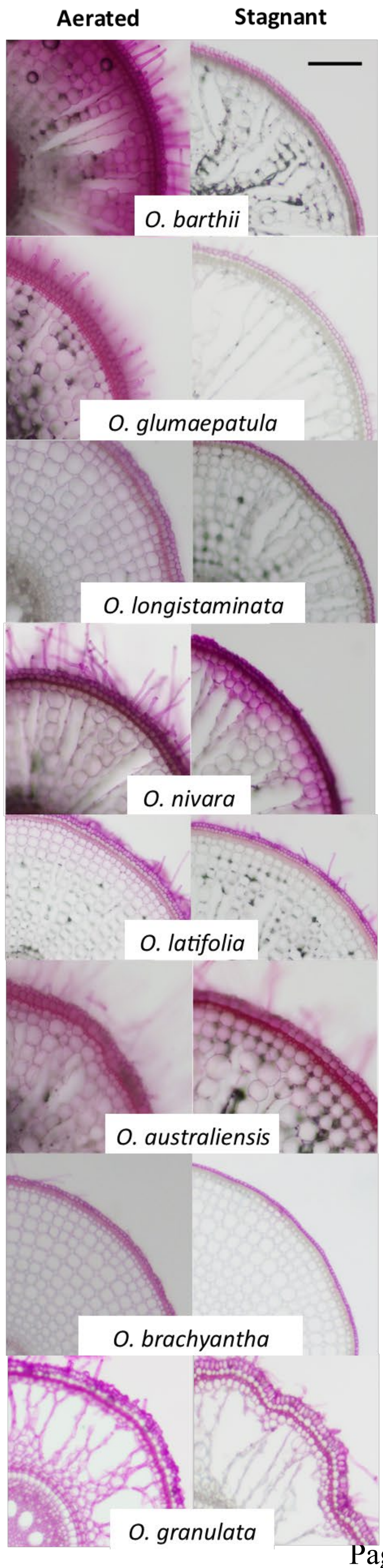
Aerated
Stagnant
O. barthii
O. glumaepatula
O. longistaminata
O. nivara
O. latifolia
O. australiensis
O. brachyantha
O. granulata

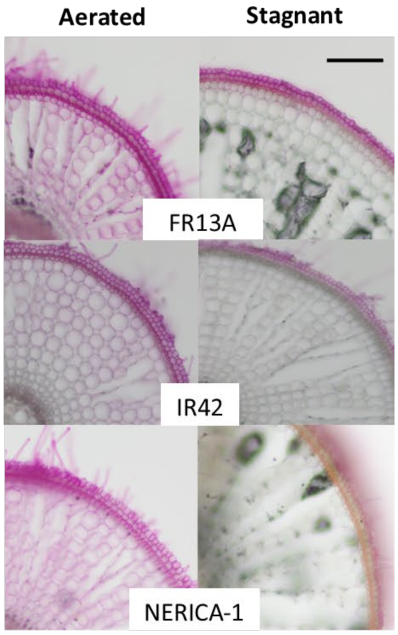
Aerated
Stagnant
FR13A
IR42
NERICA-1

30

Fig. S2.

Stained root cross-sections of 8 wild relatives of rice and 3 genotypes of *O. sativa* (FR13A, IR42 and NERICA-1) using periodic acid as an apoplastic tracer. The cross-sections were taken from a 25 mm long section representing the position 30-55 mm behind the root apex from 100-120 mm long adventitious roots. Purple colouration results from staining with Schiff's reagent that reacts with the apoplastic traces. Unstained or weakly stained tissues indicated the presence of an apoplastic barrier exterior to these tissues. Left and right columns show roots grown under aerated and stagnant conditions, respectively. Note that *O. glumaepatula*, *O. latifolia* and *O. australiensis* are also shown in Fig. 2. Scale bar is 200 µm.
